# Condition-Specific LC Retention Time Prediction: Feature-Selected QSRR Versus Pretrained Graph Isomorphism Network Transfer Learning

**DOI:** 10.3390/ijms27167164

**Published:** 2026-08-11

**Authors:** Roman Szucs, Emília Sýkorová, Iveta Boháčová, Ilaria Neri, Lucy M. Morgan, Merisa Moriarty, Melissa Hanna-Brown

**Affiliations:** 1 Department of Analytical Chemistry, Faculty of Natural Sciences, Comenius University Bratislava, Ilkovičova 6, SK-84215 Bratislava, Slovakia; sykorova132@uniba.sk (E.S.); hukelova4@uniba.sk (I.B.); 2 School of Chemistry, University College Cork, College Road, T12 YN60 Cork, Ireland; ineri@ucc.ie (I.N.); mhanna-brown@ucc.ie (M.H.-B.); 3 Pfizer Worldwide Research, Development and Medical, Sandwich CT13 9NJ, UK; lucy.morgan@pfizer.com; 4 Pfizer Worldwide Research, Development and Medical, Ringaskiddy, P43 X336 Cork, Ireland; merisa.moriarty@pfizer.com

**Keywords:** quantitative structure–retention relationships, retention time prediction, feature selection, graph isomorphism network, transfer learning, deep learning, molecular graphs, reversed-phase liquid chromatography, Mordred descriptors

## Abstract

Condition-specific liquid chromatographic retention time prediction remains challenging because retention depends on both molecular structure and experimental conditions. This study compared feature-selected quantitative structure–retention relationship (FS-QSRR) models with pretrained graph isomorphism network (GIN) transfer learning for three reversed-phase LC datasets measured under acidic, neutral, and basic conditions. Mordred descriptor-based QSRR models were developed using leakage-safe preprocessing, nested cross-validation, and feature selection. The selected FS-QSRR workflow for each dataset was then compared with pretrained GIN transfer learning using identical 100 repeated random 80/20 train–test splits. Feature selection substantially reduced descriptor dimensionality but did not consistently improve predictive accuracy over the best baseline descriptor models. Under matched validation, GIN transfer learning gave lower RMSE for all three datasets, decreasing error from 0.816 to 0.613 min under acidic conditions, from 0.868 to 0.665 min under neutral conditions, and from 1.019 to 0.876 min under basic conditions. The corresponding RMSE reductions were 24.8%, 23.4%, and 14.0%, respectively. Matched prediction error analysis showed that GIN particularly reduced the frequency and magnitude of large errors, although the improvement was more modest under basic conditions. Descriptor frequency analysis revealed condition-dependent contributions from lipophilicity, ionization-related, electronic, and topological descriptor groups. These findings support pretrained GIN transfer learning as the stronger predictive approach, while FS-QSRR remains valuable for model simplification and chemical interpretation.

## 1. Introduction

Quantitative structure–retention relationship (QSRR) modelling enables liquid chromatography (LC) retention times to be predicted directly from molecular structure. In a typical QSRR workflow, compounds with experimentally measured retention times are encoded using molecular descriptors or fingerprints, and statistical or machine learning models are trained to relate these molecular representations to retention under defined chromatographic conditions [1,2,3,4]. Such models have been applied to compound identification, retention time annotation, method development support, and reductions in experimental workload within chromatographic workflows.

Previous descriptor-based QSRR studies have shown that retention prediction can be approached using different chemometric and machine learning strategies, including partial least squares, support vector regression, tree-based models, and variable selection procedures [5,6,7,8,9]. These studies also show that predictive performance can depend strongly on the descriptor set, regression algorithm, validation design, and chromatographic system. This is particularly important for small, condition-specific LC datasets, where the number of calculated descriptors may be much larger than the number of experimentally measured compounds.

Traditional descriptor-based QSRR approaches remain attractive because they are experimentally accessible and chemically interpretable. Molecular descriptors encode physicochemical, constitutional, topological, geometrical, electronic, and molecular field information [10,11,12,13,14,15,16]. Such descriptors can often be related, at least at the descriptor-family level, to molecular properties such as hydrophobicity, polarity, ionization state, hydrogen-bonding capacity, molecular size, topology, molecular interaction fields, and electronic structure. This interpretability is particularly valuable in chromatography, where retention is governed by a combination of analyte properties and experimental variables, including mobile-phase composition, pH, gradient profile, and stationary-phase chemistry. However, descriptor-based QSRR models also face important challenges. Modern descriptor generators, such as Mordred [14], produce hundreds to thousands of candidate descriptors, many of which may be redundant, highly correlated, or only weakly relevant to retention. When the number of descriptors is large relative to the number of compounds, model overfitting, unstable descriptor selection, and limited generalizability become important concerns.

Feature selection is therefore commonly used in descriptor-based QSRR modelling to reduce dimensionality, improve model parsimony, and support chemical interpretation [17]. Simple preprocessing steps, such as removal of non-numerical descriptors, zero-variance descriptors, and highly correlated variables, can remove trivial redundancy. However, descriptor spaces may remain large even after such filtering, requiring explicit feature selection strategies. These include filter methods, embedded approaches such as regularized regression, and wrapper methods such as recursive feature elimination [17,18]. The effectiveness of feature selection is not universal and may depend on the regression algorithm, descriptor structure, dataset size, chemical domain, and chromatographic conditions [5,19]. Consequently, feature selection should be evaluated not only in terms of prediction error but also in terms of model complexity, descriptor stability, and chemical interpretability. At the same time, retention time prediction has increasingly moved toward large-scale machine learning and deep learning approaches. A major development was the METLIN small-molecule retention time dataset, which provided retention measurements for approximately 80,000 compounds acquired under a standardized reversed-phase LC-MS method [20]. This dataset enabled large-scale modelling of LC retention and subsequently became an important source dataset for deep learning and transfer learning studies. However, even large retention datasets represent only a part of chemical and chromatographic space and are tied to specific experimental conditions. Their applicability to new analyte classes, mobile phases, stationary phases, or pH conditions therefore cannot be assumed without local validation.

Deep learning models have been explored using several molecular representations, including descriptors, fingerprints, SMILES-based representations, and molecular graphs [21,22,23]. Fedorova et al. evaluated neural network approaches for reversed-phase LC retention prediction and showed that transfer learning from METLIN-SMRT could improve prediction on smaller target datasets in several cases [24]. Importantly, they also concluded that transfer learning still required local datasets of substantial size, approximately at least 100–150 compounds, to be effective. Osipenko et al. investigated transfer learning for small-molecule retention prediction and emphasized that transfer across chromatographic systems remains challenging because relationships between retention times measured under different experimental conditions are not always straightforward [25]. Kwon et al. directly addressed retention prediction from small target datasets by pretraining a GIN on METLIN-SMRT and fine-tuning it on individual target chromatographic datasets containing 38–532 compounds [26]. Their work is particularly relevant to condition-specific LC modelling because it treats each chromatographic system as a local target domain requiring its own calibration data.

Recent pharmaceutical studies further illustrate both the promise and the constraints of large-data and transfer learning approaches. Beck and co-workers developed retention time models using tens of thousands of proprietary pharmaceutical analytes and showed that strong predictive performance could be achieved when the training data adequately represented the chemical descriptor space of interest [27]. In a subsequent low-data transfer learning study, Beck et al. demonstrated that a GIN pretrained on a larger internal dataset could be fine-tuned using only 80 local training analytes, substantially outperforming commercial QSRR models for that application [28]. These studies show that transfer learning can provide major predictive benefits, but they also reinforce a central point: local, condition-specific retention data remain necessary. The current challenge is therefore not simply whether large datasets and transfer learning are useful; they clearly are. Rather, the practical scientific question is how different modelling strategies perform when a condition-specific LC retention dataset is available for the chromatographic system of interest. In such cases, two complementary modelling routes can be considered. Descriptor-based QSRR offers transparency, lower implementation complexity, and chemical interpretation through selected molecular descriptors. Pretrained GIN transfer learning offers a modern predictive benchmark that may exploit structural representations learned from much larger retention datasets. A direct comparison between these two approaches under identical validation conditions is therefore needed.

In this study, we compare feature-selected QSRR and pretrained GIN transfer learning for condition-specific LC retention time prediction. Three experimentally generated reversed-phase LC datasets were used, corresponding to acidic, neutral, and basic mobile-phase conditions and different stationary-phase chemistries. First, a descriptor-based QSRR workflow using Mordred descriptors was developed, including baseline modelling, leakage-safe preprocessing, feature selection benchmarking, nested model selection, and repeated external validation. Second, the selected QSRR models were benchmarked against the pretrained GIN transfer learning approach of Kwon et al. [26], using the same 100 repeated 80/20 train–test splits. This design enabled direct comparison of predictive accuracy, relative error, and the frequency of large prediction errors. Finally, matched prediction error analysis was used to determine whether transfer learning improved predictions uniformly or primarily reduced high-error outliers, while descriptor frequency analysis was used to retain the interpretive advantage of QSRR across the chromatographic systems. The study was not intended as a comprehensive benchmark of global retention time prediction models but as a matched comparison of two modelling strategies adapted separately to the same condition-specific chromatographic datasets.

## 2. Results and Discussion

### 2.1. Baseline Model Performance

To establish a reference point for evaluating the effect of feature selection, a range of regression algorithms was first assessed without explicit feature selection. All models were evaluated using the nested cross-validation framework described above, consisting of 10 outer folds repeated with 10 random seeds, with hyperparameter optimization performed within the inner loop for each outer training split. This design ensured that performance estimates reflected out-of-sample predictive ability and were not biased by model tuning.

The baseline results showed that predictive performance varied across both regression algorithm and chromatographic condition (Table 1).

For dataset1, corresponding to the acidic chromatographic condition, the lowest baseline error was obtained with Extra Trees regression, with RMSE = 0.759 ± 0.216 min, MAE = 0.576 ± 0.163 min, and R^2^ = 0.656 ± 0.158. Elastic Net was the next best-performing model, while SVR, PLS, Random Forest, and KNN generally produced higher errors.

For dataset2, corresponding to the neutral condition, Extra Trees again gave the lowest RMSE, although the difference from SVR with an RBF kernel was small. Extra Trees achieved RMSE = 0.781 ± 0.178 min, MAE = 0.617 ± 0.145 min, and R^2^ = 0.616 ± 0.486, compared with RMSE = 0.794 ± 0.208 min for SVR-RBF. Several nonlinear models therefore performed similarly for dataset2, indicating that the neutral dataset did not strongly favour a single regression approach.

For dataset3, corresponding to the basic condition, Elastic Net gave the best baseline performance, with RMSE = 0.916 ± 0.259 min, MAE = 0.711 ± 0.212 min, and R^2^ = 0.639 ± 0.223. Nonlinear methods, including SVR-RBF and tree-based models, showed higher errors. This suggests that, for the basic dataset, a regularized linear model captured the dominant descriptor–retention relationships more effectively than the nonlinear baseline models evaluated here.

Overall, the baseline assessment confirmed that no single regression algorithm was uniformly optimal across all chromatographic conditions. Extra Trees performed best for the acidic and neutral datasets, whereas Elastic Net performed best for the basic dataset. These results provided the reference point for evaluating whether explicit feature selection could improve model parsimony and predictive performance.

### 2.2. Effect of Feature Selection

To evaluate the effect of descriptor reduction on QSRR model performance, feature selection strategies representing filter-based, embedded, and wrapper-based approaches were investigated. These included SelectKBest followed by Elastic Net, embedded Elastic Net, RFE with Elastic Net, RFE with Extra Trees, and RFE-Linear SVR followed by SVR-RBF. In the RFE-Linear SVR/SVR-RBF workflow, recursive feature elimination was performed using a linear SVR estimator to rank and select descriptors, whereas the final predictive model was an SVR with a radial basis function kernel.

All feature selection procedures were implemented within the nested cross-validation framework. Feature selection parameters and model hyperparameters were optimized jointly by exhaustive grid search within the inner cross-validation loop. Descriptor preprocessing, including removal of zero-variance and highly correlated descriptors, was performed within each outer training fold. This ensured that descriptor filtering, feature selection, and model tuning were based only on the training data for each split. The results of the feature selection assessment are summarized in Table 2.

Overall, feature selection did not produce a uniform improvement in predictive accuracy relative to the best baseline models. Instead, its main benefit was the reduction in descriptor dimensionality while maintaining broadly comparable predictive performance. This indicates that the Mordred descriptor space contained substantial redundancy and that retention time prediction could often be achieved using considerably smaller descriptor subsets.

Several factors may explain why explicit feature selection did not consistently reduce prediction error. First, the strongest baseline algorithms already controlled model complexity to some extent. Elastic Net performs coefficient shrinkage and embedded variable selection, whereas Extra Trees combines randomized descriptor selection with ensemble averaging and can therefore remain effective in high-dimensional, redundant descriptor spaces. Explicit descriptor reduction consequently had limited opportunity to improve these already regularized or ensemble-based models. Second, although the Mordred descriptor space contained substantial redundancy, retention-relevant information may have been distributed across groups of correlated or partially interchangeable descriptors. Feature selection could therefore remove many variables while retaining a smaller subset carrying similar predictive information, resulting in improved parsimony but little change in accuracy. More aggressive reduction could also discard complementary information. Finally, the relatively small sizes of the three condition-specific datasets, comprising 105–117 compounds, limited the amount of information available for distinguishing among closely performing descriptor subsets and may have increased variability in descriptor selection between training folds. These factors provide plausible explanations for why feature selection primarily simplified the models rather than consistently improving their predictive performance.

For dataset1, corresponding to the acidic chromatographic condition, the best feature-selected workflow was RFE-Extra Trees/Extra Trees. This approach gave RMSE = 0.770 ± 0.209 min, MAE = 0.585 ± 0.151 min, and R^2^ = 0.639 ± 0.178 while selecting an average of only 14.8 descriptors. Although the predictive performance was similar to, rather than clearly better than, the best baseline model, the large reduction in descriptor number makes this workflow attractive from the perspective of model parsimony and interpretability.

For dataset2, corresponding to the neutral chromatographic condition, the best feature-selected workflow was RFE-Linear SVR/SVR-RBF, with RMSE = 0.792 ± 0.216 min, MAE = 0.604 ± 0.154 min, and R^2^ = 0.606 ± 0.387. RFE-Extra Trees/Extra Trees gave very similar performance, with RMSE = 0.794 ± 0.192 min, MAE = 0.629 ± 0.158 min, and R^2^ = 0.599 ± 0.477. The differences between the best-performing feature selection workflows were therefore small for dataset2, suggesting that several reduced descriptor representations captured similar predictive information. 

For dataset3, corresponding to the basic chromatographic condition, embedded Elastic Net gave the lowest prediction error among the feature selection workflows. It achieved RMSE = 0.916 ± 0.259 min, MAE = 0.711 ± 0.212 min, and R^2^ = 0.639 ± 0.223, with an average of 68.9 descriptors having non-zero coefficients. Its predictive performance was the same as that of the baseline Elastic Net at the reported precision. This equivalence was expected because feature selection in the embedded workflow was intrinsic to the fitted Elastic Net model rather than being implemented as a separate feature selection step followed by refitting a reduced model. Thus, the embedded analysis did not improve predictive accuracy beyond the baseline Elastic Net, but it made the sparsity of the fitted model explicit by identifying the descriptors retained with non-zero coefficients.

Across the three datasets, the optimal feature selection strategy was condition-dependent. RFE-Extra Trees/Extra Trees was most effective for dataset1, RFE-Linear SVR/SVR-RBF was most effective for dataset2, and embedded Elastic Net was most effective for dataset3. Filter-based SelectKBest generally retained much larger descriptor subsets, particularly for dataset1 and dataset3, without providing a corresponding improvement in predictive accuracy. In contrast, RFE-based and embedded approaches produced more compact models while maintaining similar predictive performance.

Overall, these results indicate that feature selection was most valuable as a strategy for model simplification and interpretation rather than as a universal route to lower prediction error. The condition-dependent differences in the best-performing feature selection workflows also show that descriptor reduction should be optimized separately for each chromatographic system rather than assumed to transfer unchanged across conditions.

As an additional control for chance correlation, Y-randomization was performed for the selected dataset1 FS-QSRR workflow, RFE-Extra Trees/Extra Trees. In the dedicated Y-randomization evaluation, the model using the true retention time response gave pooled outer-fold RMSE = 0.781 min, MAE = 0.582 min, and R^2^ = 0.711. Across 100 independently randomized response vectors, the corresponding mean values were RMSE = 1.493 ± 0.025 min, MAE = 1.252 ± 0.023 min, and R^2^ = −0.056 ± 0.035. None of the 100 randomized response models achieved an RMSE or MAE as low as, or an R^2^ as high as, the true response model. The empirical permutation probability was therefore 0.0099 for all three metrics. These results provide evidence that the predictive performance of the selected dataset1 FS-QSRR workflow was not attributable to chance correlation. The complete results are provided in Table S5.

### 2.3. Feature Selection Frequency and Descriptor Interpretation

To examine whether the feature selection workflows identified chemically meaningful descriptor patterns, descriptor selection frequencies were analysed across the nested cross-validation runs. Because individual descriptor selection can depend on the feature selection algorithm and on correlations among descriptors, interpretation was based primarily on descriptors that were selected repeatedly across multiple feature selection workflows rather than on the descriptor list from a single model. A descriptor was considered robust when it was selected in at least 50% of outer evaluations in at least three of the five feature selection workflows. This criterion was used as a predefined operational definition of robustness rather than as a statistically optimized threshold. The 50% requirement ensured that a descriptor was retained in the majority of outer evaluations within a workflow, while the requirement for selection in at least three of the five workflows required agreement across the majority of the investigated feature selection approaches. The combined criterion was intended to exclude descriptors selected only sporadically or by a single method. The resulting interpretation was therefore based primarily on recurring descriptor families and cross-method convergence rather than on the exact membership of a threshold-defined descriptor list.

Selected descriptors were interpreted mainly at the descriptor-family level. Descriptor-family assignments were based on the Mordred descriptor documentation [29] and the original Mordred software publication [14]. This level of interpretation is appropriate because many Mordred descriptors are abstract numerical encodings of molecular structure rather than direct measurements of a single physicochemical property. Some descriptors are straightforward to interpret, such as nAcid and nBase, which count acidic and basic functional groups. Others summarize calculated physicochemical properties, such as SLogP, corresponding to Wildman–Crippen logP, and FilterItLogS, corresponding to a calculated aqueous solubility descriptor. Atom-type descriptors, including AMID_N, AMID_h, NaaN, NssO, and related variables, encode specific atomic or substructural environments. For example, NaaN represents the count of aromatic ring nitrogen atoms belonging to the aaN atom-type class. Other descriptor families encode how atomic properties are distributed through the molecular graph, including autocorrelation descriptors such as AATSC, ATSC, MATS, and GATS. Surface area descriptors such as PEOE_VSA and EState_VSA summarize molecular surface contributions grouped by partial charge or electrotopological state, whereas BCUT descriptors are eigenvalue-based descriptors derived from Burden matrices weighted by atomic properties such as atomic number, charge, or polarizability. Ring count and Chi descriptors, including n6aHRing, n10FaRing, nSpiro, nBondsT, and Xc-4dv, provide information on molecular topology, ring systems, branching, and connectivity. The following interpretation therefore focuses on recurring descriptor groups and their chemical context rather than on isolated descriptors as direct mechanistic variables.

For the acidic dataset (dataset1), the robust descriptor pattern combined basic and nitrogen-containing structural features with lipophilicity and charge/surface descriptors. The most consistently selected descriptors were nBase, AMID_N, and SLogP, which were frequent in all five feature selection workflows. nBase was selected with a frequency of 1.00 in all methods, indicating that the presence of basic functional groups was a stable descriptor signal under the acidic condition. AMID_N, an averaged molecular identity descriptor focused on nitrogen atoms, was also highly stable, with a mean selection frequency of 0.984. SLogP, a calculated lipophilicity descriptor, showed a mean selection frequency of 0.916, indicating that hydrophobicity-related information remained important even under the acidic mobile-phase condition. Additional frequently selected descriptors included PEOE_VSA13, MATS6i, NaaN, SdssC, n6aHRing, and ETA_dEpsilon_D. These descriptors represent charge-binned molecular surface area, autocorrelation of atomic properties, aromatic nitrogen environments, carbon atom-type environments, aromatic hetero-ring count, and electronic descriptor information. Overall, this pattern suggests that retention under the acidic condition was associated with a combination of basic or ionizable functionality, nitrogen-containing structural motifs, lipophilicity, charge-distributed surface area, and electronic/topological descriptors.

The neutral dataset (dataset2) showed a more mixed descriptor profile. ETA_dEpsilon_D was the most stable descriptor, being frequent in all five feature selection workflows, with a mean selection frequency of 0.860. This descriptor belongs to the extended topochemical atom descriptor family and reflects electronic structure information. Lipophilicity and solubility-related descriptors also remained important: SLogP was frequent in four methods, with a mean selection frequency of 0.880, while FilterItLogS was frequent in four methods, with a mean selection frequency of 0.692. AMID_N was also frequent in four methods, indicating that nitrogen-related structural information remained relevant. Additional robust descriptors included nX, AMID_h, ATS2d, ATSC0i, n6Ring, BCUTZ-1l, MDEC-23, VE2_A, and PEOE_VSA6. These descriptors include halogen count, heteroatom-focused molecular identity descriptors, autocorrelation descriptors, ring count descriptors, BCUT eigenvalue descriptors weighted by atomic number, graph distance or connectivity descriptors, and charge-binned surface area descriptors. The neutral C18 system was therefore associated with a descriptor profile that combined lipophilicity/solubility, heteroatom composition, electronic structure, ring/topological information, and surface area descriptors.

For the basic dataset (dataset3), obtained using the BEH phenyl-hexyl column, the selected descriptor profile was broader and included several highly stable descriptor groups. The most robust descriptor was nAcid, which was selected in all folds by all five feature selection workflows. This indicates that acidic functionality was the most stable descriptor signal under the basic mobile-phase condition. ETA_dEpsilon_D was also frequent in all five methods, with a mean selection frequency of 0.918, supporting the contribution of electronic descriptor information. Additional robust descriptors included AATSC1c, FilterItLogS, nSpiro, n10FaRing, AATSC0c, Xc-4dv, EState_VSA4, BCUTc-1l, ATSC3dv, BCUTZ-1l, NssO, PEOE_VSA2, and nBondsT. These descriptors represent several different structural and physicochemical categories: charge-based autocorrelation descriptors, predicted solubility, spiroatom and fused aromatic ring counts, Chi cluster connectivity descriptors weighted by valence electrons, electrotopological-state surface area descriptors, BCUT eigenvalue descriptors, oxygen atom-type environments, charge-binned surface area descriptors, and multiple bond counts. Lipophilicity/solubility-related descriptors were also retained, with FilterItLogS frequent in all five methods and SLogP frequent in three methods. This descriptor pattern suggests that retention in dataset3 was associated with a combination of ionization-related, electronic, topological, ring system, and lipophilicity/solubility descriptors.

Overall, the feature frequency analysis indicates that lipophilicity-related information remained relevant across all three chromatographic conditions, but it appeared together with different co-selected descriptor groups in each dataset. Dataset1 combined lipophilicity with basic and nitrogen-containing descriptors, dataset2 showed a mixed lipophilicity/solubility, heteroatom, electronic, and topological descriptor profile, and dataset3 combined lipophilicity/solubility descriptors with a particularly strong ionization-related signal and multiple electronic, ring system, and topological descriptors. These findings support a condition-dependent descriptor profile for retention time prediction. Together with the differences in the optimal feature selection and regression workflows, this condition dependence indicates that a single fixed FS-QSRR descriptor basis should not be assumed to transfer unchanged across chromatographic conditions.

At the same time, descriptor interpretation should be treated cautiously. Some descriptors, such as nBase in dataset1 and nAcid in dataset3, were highly stable across feature selection methods and are chemically straightforward to interpret. Others, including SLogP in dataset3 and many autocorrelation, BCUT, VSA, and Chi descriptors, showed method-dependent selection behaviour or represent abstract mathematical summaries of molecular structure. Descriptor selection frequency reflects the reproducibility with which a descriptor was retained across the investigated feature selection workflows; it does not measure the magnitude or direction of its contribution to prediction and should not be interpreted as a direct measure of feature importance or causality. For this reason, the descriptor analysis is best interpreted at the level of recurring descriptor groups and cross-method convergence, rather than as definitive evidence for isolated mechanistic effects. The supporting descriptor frequency data are provided in the Supporting Information as a consolidated table of robust and manuscript-discussed descriptors, including descriptor-family assignments, short descriptor interpretations, mean selection frequencies, workflow-specific selection frequencies, and the feature selection workflows in which each descriptor was repeatedly selected (Table S3).

### 2.4. Direct Comparison with Pretrained GIN Transfer Learning

The best feature-selected QSRR workflow for each dataset was evaluated against pretrained GIN transfer learning using identical repeated 80/20 train–test splits. For each of the 100 random splits, the descriptor-based QSRR model and the pretrained GIN model were trained or fine-tuned on the same local training compounds and evaluated on the same held-out test compounds. This design enabled a direct matched comparison between descriptor-based and graph-based modelling under matched validation conditions.

**Figure 1:**
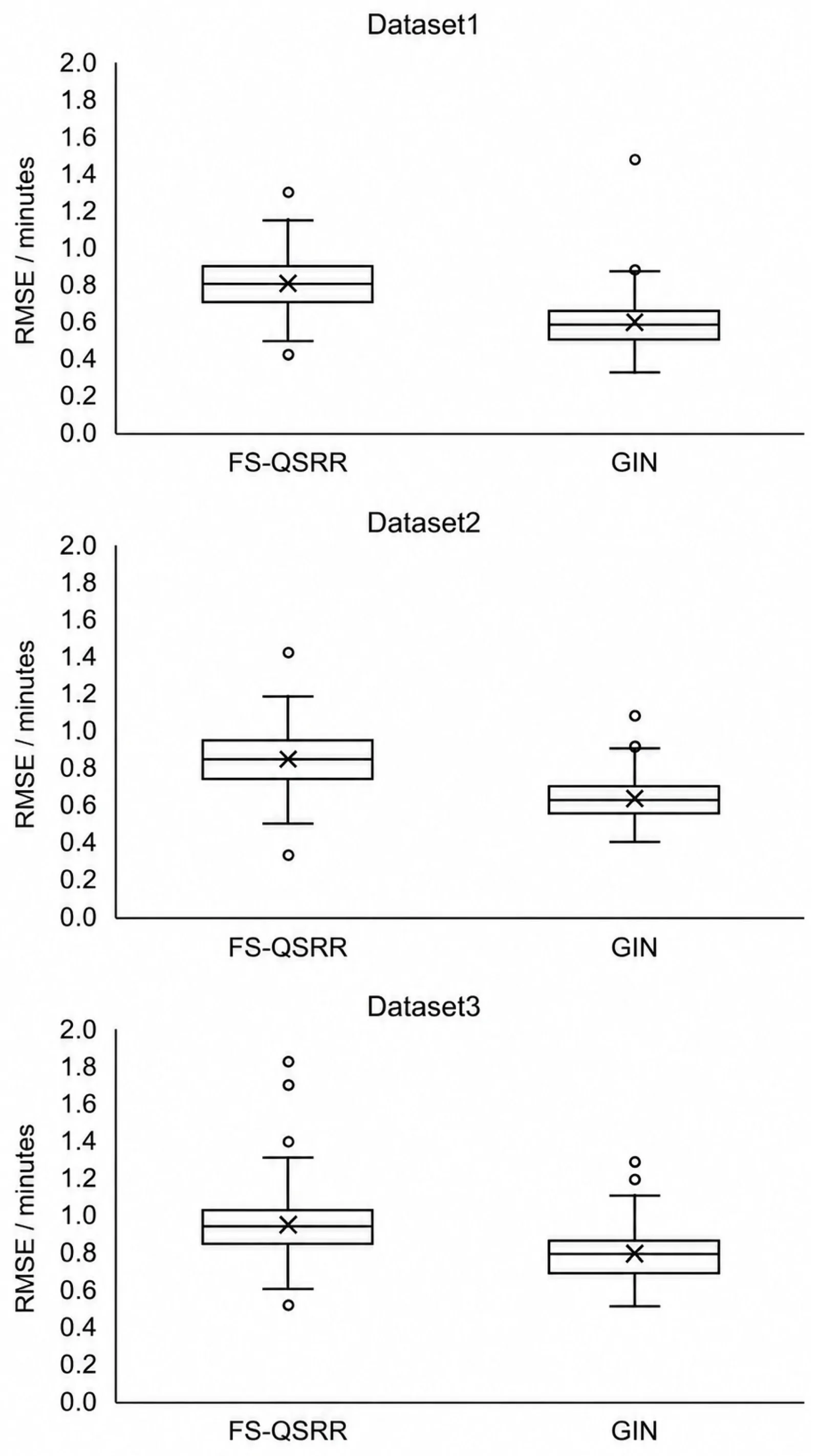
Split-level root mean squared error (RMSE) distributions for the selected feature-selected quantitative structure–retention relationship (FS-QSRR) workflow and pretrained graph isomorphism network (GIN) transfer learning across 100 matched repeated random 80/20 train–test splits. The three panels represent dataset1 under acidic conditions, dataset2 under neutral conditions, and dataset3 under basic conditions. The vertical axis shows RMSE in minutes; lower values indicate better predictive performance. Within each box plot, the horizontal central line represents the median, the box represents the interquartile range, the whiskers extend to the most extreme values within 1.5 times the interquartile range, individual circles represent splits outside the whiskers, and the cross represents the mean RMSE across the 100 splits. FS-QSRR and GIN were evaluated using identical training and test compounds within each matched split.

The resulting split-level RMSE distributions are shown in Figure 1. Because the same 100 random 80/20 train–test splits were used for both modelling approaches, each FS-QSRR result has a directly matched GIN result for the same dataset and split. Across all three datasets, pretrained GIN transfer learning shifted the RMSE distribution toward lower values relative to the corresponding best FS-QSRR workflow. The separation between the two approaches was most pronounced for dataset1 and dataset2, whereas dataset3 showed a smaller but still evident improvement.

For dataset1, dataset2, and dataset3, the 80/20 split design produced 21, 24, and 23 test compounds per split, respectively. Across 100 repeated random splits, this resulted in 2100, 2400, and 2300 held-out test predictions for each modelling approach. The same test compounds were used for FS-QSRR and GIN within each split, allowing prediction errors to be matched directly by compound identifier and split number. Although compounds could appear in the test set in more than one split, each prediction was generated from a model trained without that compound in the corresponding split.

Across all three chromatographic systems, pretrained GIN transfer learning produced lower prediction errors than the best feature-selected QSRR workflow (Table 3). For dataset1, RMSE decreased from 0.816 ± 0.141 min for RFE-Extra Trees/Extra Trees to 0.613 ± 0.143 min for GIN transfer learning. MAE decreased from 0.604 ± 0.102 to 0.479 ± 0.119 min, and mean R^2^ increased from 0.659 ± 0.105 to 0.802 ± 0.111.

A similar improvement was observed for dataset2. The best feature-selected QSRR workflow, RFE-Linear SVR/SVR-RBF, gave RMSE = 0.868 ± 0.162 min and MAE = 0.664 ± 0.126 min. In comparison, pretrained GIN transfer learning gave RMSE = 0.665 ± 0.111 min and MAE = 0.518 ± 0.087 min. The corresponding mean R^2^ increased from 0.618 ± 0.159 to 0.773 ± 0.102.

For dataset3, the improvement was smaller but remained consistent. Embedded Elastic Net gave RMSE = 1.019 ± 0.187 min, MAE = 0.766 ± 0.134 min, and R^2^ = 0.619 ± 0.169. Pretrained GIN transfer learning reduced RMSE to 0.876 ± 0.126 min and MAE to 0.687 ± 0.107 min, with R^2^ increasing to 0.721 ± 0.099.

Expressed as relative changes, GIN reduced RMSE by 24.8%, 23.5%, and 14.0% for dataset1, dataset2, and dataset3, respectively. MAE decreased by 20.7%, 22.0%, and 10.3%, respectively. Median relative error was also lower for GIN in all three datasets, decreasing from 8.988% to 7.773% for dataset1, from 8.656% to 6.892% for dataset2, and from 7.950% to 7.356% for dataset3.

These results indicate that pretrained GIN transfer learning provided the strongest predictive performance under matched repeated 80/20 validation. The improvement was most pronounced for dataset1 and dataset2, while the difference between FS-QSRR and GIN was more modest for dataset3. This suggests that the benefit of transfer learning was condition-dependent but consistently favourable across the three chromatographic systems evaluated.

The observed advantage of GIN should be interpreted as the performance of the complete pretrained graph-based workflow rather than as evidence for the superiority of any single modelling component. The two approaches differ simultaneously in molecular representation, model architecture, and prior training. FS-QSRR uses calculated Mordred descriptor vectors together with conventional regression and feature selection methods, whereas GIN operates on atom- and bond-level molecular graphs and was pretrained on the much larger METLIN-SMRT retention dataset before local fine-tuning. Consequently, the present comparison does not distinguish whether the improvement arose primarily from the molecular graph representation, the nonlinear GIN architecture, the information transferred from pretraining, or an interaction among these factors.

Several mechanisms may plausibly contribute. Graph representations retain explicit local atom, bond, ring, charge, and connectivity information, whereas descriptor-based models use a fixed set of calculated molecular summaries. Pretraining may additionally provide transferable structural representations learned from a chemical space substantially larger than the local chromatographic datasets. The GIN architecture may also capture nonlinear combinations of structural features that differ from those learned by the regression algorithms evaluated in the FS-QSRR workflow. Isolating these effects would require a controlled ablation study, for example, comparisons with a randomly initialized GIN trained only on each local dataset, pretrained models with different degrees of parameter freezing, and fingerprint- or descriptor-based models evaluated using comparable neural network architectures. Such experiments were beyond the scope of the present study, whose objective was to compare the two complete modelling strategies under identical validation conditions.

### 2.5. Matched Prediction Error Analysis

The direct comparison in Section 2.4 showed that pretrained GIN transfer learning reduced average prediction errors relative to the best feature-selected QSRR workflows. However, average metrics such as RMSE and MAE do not fully describe how the improvement arises. A lower average error may reflect a small improvement across most predictions, a reduction in a smaller number of large errors, or a combination of both effects. To examine this more directly, a matched prediction error analysis was performed.

Because both modelling approaches were evaluated using identical repeated 80/20 train–test splits, each prediction from the feature-selected QSRR model could be paired with the corresponding prediction from the GIN model for the same compound in the same split. For dataset1, dataset2, and dataset3, the repeated split design generated 2100, 2400, and 2300 matched held-out predictions, respectively. Although individual compounds could appear in more than one test split, each prediction was generated from a model trained without that compound in the corresponding split.

The distribution of absolute errors is summarized in Table 4. In addition to the median absolute error, the 75th, 90th, and 95th percentiles were included to distinguish between typical, moderate, and high-error predictions. This was important because the central part of the error distribution did not always differ strongly between the two approaches, whereas larger differences were observed in the upper tail.

**Figure 2:**
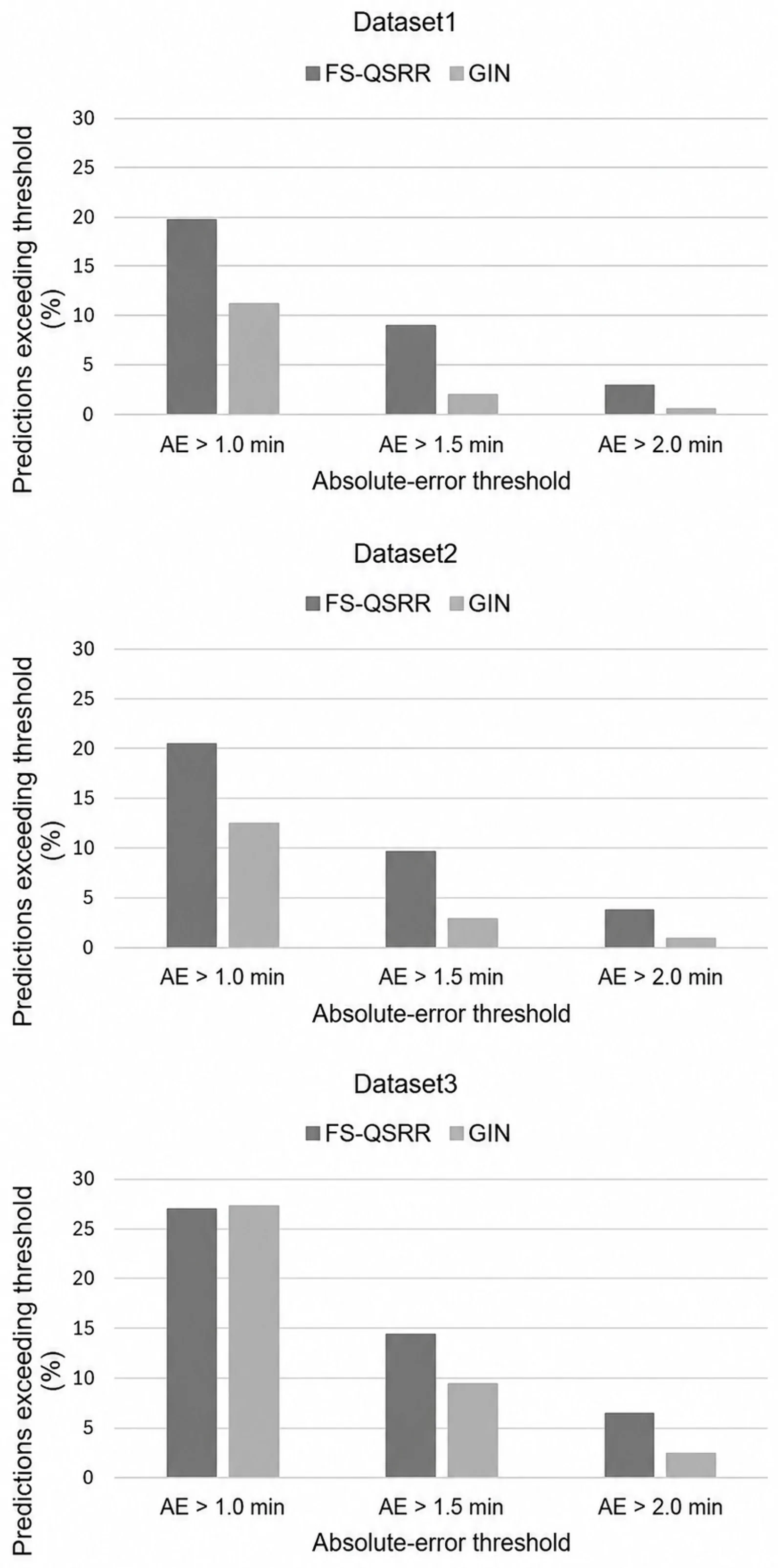
Absolute error tail comparison for the selected feature-selected quantitative structure–retention relationship (FS-QSRR) workflows and pretrained graph isomorphism network (GIN) transfer learning. The three panels represent dataset1, dataset2, and dataset3. The horizontal axis shows the absolute error thresholds AE > 1.0, AE > 1.5, and AE > 2.0 min, where AE is the absolute difference between the experimental and predicted retention times. The vertical axis shows the percentage of matched held-out predictions exceeding each threshold. Dark bars represent FS-QSRR and light bars represent GIN. The percentages were calculated from 2100, 2400, and 2300 matched held-out predictions for dataset1, dataset2, and dataset3, respectively, generated across the 100 repeated random 80/20 train–test splits. Lower percentages indicate fewer large prediction errors.

The proportions of predictions exceeding predefined absolute error thresholds are shown in Figure 2. This representation directly visualizes the upper error tail of the prediction error distribution and highlights the reduction in large prediction errors obtained with pretrained GIN transfer learning, particularly for dataset1 and dataset2.

For dataset1, the median absolute error decreased from 0.419 min for FS-QSRR to 0.378 min for GIN. The difference became more pronounced at higher error percentiles: the 75th percentile decreased from 0.848 to 0.688 min, the 90th percentile from 1.427 to 1.032 min, and the 95th percentile from 1.739 to 1.246 min. The proportion of predictions with absolute error greater than 1.0 min decreased from 19.7% to 11.2%, while errors greater than 2.0 min decreased from 3.0% to 0.6%.

A similar pattern was observed for dataset2. The median absolute error decreased from 0.516 to 0.419 min, while the 75th percentile decreased from 0.898 to 0.741 min. Larger differences were again observed in the upper tail, with the 90th percentile decreasing from 1.471 to 1.060 min and the 95th percentile decreasing from 1.893 to 1.272 min.

For dataset3, the difference between the two approaches was smaller in the central part of the distribution. The median absolute error decreased only slightly, from 0.568 to 0.546 min, and the 75th percentile was almost unchanged: 1.058 min for FS-QSRR and 1.054 min for GIN. However, GIN still reduced the upper tail of the error distribution. The 90th percentile decreased from 1.739 to 1.473 min, the 95th percentile decreased from 2.176 to 1.766 min, and the proportion of predictions with absolute error greater than 2.0 min decreased from 6.5% to 2.4%. The proportion of errors greater than 1.0 min was similar for the two approaches: 27.0% for FS-QSRR and 27.3% for GIN.

The paired split-level analysis confirmed that the improvement with GIN was consistent across repeated train–test partitions. GIN produced lower RMSE than FS-QSRR in 93%, 89%, and 85% of the splits for dataset1, dataset2, and dataset3, respectively; lower MAE in 91%, 89%, and 72% of the splits; and higher $R^{2}$ in 93%, 89%, and 85% of the splits. The mean paired RMSE differences, calculated as GIN minus FS-QSRR, were −0.203, −0.204, and −0.143 min for dataset1, dataset2, and dataset3, respectively.

Two-sided paired Wilcoxon signed-rank tests showed statistically significant differences between GIN and FS-QSRR for RMSE, MAE, and $R^{2}$ in all three datasets. All nine comparisons remained statistically significant after Holm adjustment for multiple testing, with adjusted *p*-values ranging from $1.35\times 10^{-14}$ to $2.16\times 10^{-7}$. These results support the consistency of the improved GIN performance across the matched repeated train–test splits. The difference was most pronounced for dataset1 and dataset2 and more modest for dataset3, particularly for MAE. Complete statistical results are provided in Supplementary Table S4.

Because compounds were reused across repeated random splits, the split-level observations were not fully independent. The statistical tests were therefore interpreted as evidence of consistent paired model differences within the repeated validation procedure rather than as evidence obtained from 100 independent experiments.

Overall, the matched error analysis indicates that pretrained GIN transfer learning improved retention time prediction in two related ways. First, it reduced average error across repeated validation splits. Second, it reduced the frequency and magnitude of larger prediction errors, especially for dataset1 and dataset2. For dataset3, the central and moderate-error regions of the distributions were similar between the two approaches, but GIN still reduced the most severe errors. This suggests that the advantage of transfer learning was not simply a uniform shift in all predictions but was partly driven by improved control of the upper error tail.

Experimental retention times obtained under controlled LC conditions are generally substantially more repeatable than the prediction errors achieved by current retention time prediction models. The practical adequacy of these predictions should therefore be judged according to the intended application; the present accuracy may already provide useful supporting evidence for structural elucidation, reductions in false positive identifications, and assessment of the likely chromatographic behaviour of hypothetical impurities, although further improvements will be required for broader applicability.

### 2.6. Applicability Domain and Study Limitations

A formally defined applicability domain is an important element in the validation and interpretation of QSPR/QSAR models. In the present study, however, the primary objective was to compare FS-QSRR and pretrained GIN transfer learning within three small, condition-specific chromatographic datasets rather than to develop fixed models intended for unrestricted prediction. Descriptor preprocessing, feature selection, and hyperparameter optimization were repeated within each validation split. Consequently, the reported results were generated from multiple split-specific models rather than from one final model with a single fixed descriptor representation and formally defined applicability domain.

The validation results should therefore be interpreted as estimates of predictive performance within the chemical space represented by each dataset and under the corresponding chromatographic conditions. The repeated random train–test splits assess interpolation among compounds drawn from the same local dataset, but they do not establish reliable extrapolation to structurally dissimilar compounds or to different chromatographic systems. In particular, the models should not be assumed to transfer unchanged across different mobile-phase pH values, stationary-phase chemistries, gradient conditions, or analyte classes.

The absence of a formal applicability-domain analysis is therefore a limitation of the present study. Future work should define the domain of each final model using complementary measures, such as molecular structure similarity and distance within the selected descriptor or learned representation space, and should evaluate predictions using chemically distinct external compounds. Such analysis would allow individual predictions to be identified as interpolation within, or extrapolation beyond, the represented chemical and chromatographic domain.

The relatively small sizes of the three datasets, comprising 105–117 compounds, represent an additional limitation because they restrict the precision with which predictive performance, descriptor selection stability, and model generalizability can be estimated. At the same time, datasets of this scale reflect the practical reality of chromatographic modelling. Generating consistent retention data under a single, carefully controlled chromatographic method is resource-intensive, and large condition-specific datasets are rarely available to individual laboratories. The present study therefore evaluates FS-QSRR and transfer learning in a realistic small-data setting in which these approaches are likely to be applied. The use of 100 repeated matched train–test splits does not eliminate the limitations imposed by dataset size, but it provides a more informative assessment of performance variability than a single train–test partition.

The investigation was restricted to three reversed-phase LC datasets representing acidic, neutral, and basic conditions. Although these datasets allowed condition-dependent modelling behaviour to be compared, they do not represent the full diversity of chromatographic separations. Broader generalization would require evaluation across additional stationary phases, mobile-phase compositions, gradients, pH values, and analyte classes. Furthermore, the descriptor-based analysis relied on Mordred descriptors. Although Mordred provides a broad range of constitutional, physicochemical, electronic, and topological descriptors, alternative molecular representations, including fingerprints, three-dimensional descriptors, or chromatographically oriented descriptors, could produce different feature selection and modelling outcomes.

The repeated held-out predictions constitute internal, or pseudo-external, validation rather than validation using a completely independent set of newly measured compounds. The three datasets could not be used as external validation sets for one another because each represented a different chromatographic condition and therefore a different retention prediction problem. Finally, chemical interpretation is necessarily limited for mathematically abstract descriptors such as BCUT, autocorrelation, Chi, and VSA descriptors. Their repeated selection indicates statistical association and reproducibility across the investigated workflows, but it does not establish causality or a specific chromatographic retention mechanism.

## 3. Materials and Methods

### 3.1. Reversed-Phase Liquid Chromatography

All experiments were performed using an Agilent 1290 Infinity UHPLC system (Agilent Technologies, Waldbronn, Germany) equipped with a diode array detector, autosampler, and column thermostat. An Agilent 6550i quadrupole time-of-flight mass spectrometer (Agilent Technologies, Santa Clara, CA, USA) was used to track chromatographic peaks. Chromatographic data were collected and processed using MassHunter Workstation LC/MS data acquisition software, version B.09.00 (Agilent Technologies, Santa Clara, CA, USA). Chromatographic retention times used for QSRR model development were obtained under three distinct liquid chromatographic conditions corresponding to acidic, neutral, and basic mobile-phase environments, referred to as dataset1, dataset2, and dataset3, respectively. The experimental conditions were selected to probe the influence of mobile-phase pH, mobile-phase composition, and stationary-phase chemistry on retention behaviour and descriptor relevance.

Separations were performed using Waters Acquity UHPLC columns (Waters Corporation, Milford, MA, USA). Dataset1 was acquired using an Acquity UHPLC BEH Shield RP18 column (2.1 mm i.d. × 100 mm, 1.7 μm), dataset2 using an Acquity UHPLC BEH C18 column (2.1 mm i.d. × 100 mm, 1.7 μm), and dataset3 using an Acquity UHPLC BEH phenyl-hexyl column (2.1 mm i.d. × 100 mm, 1.7 μm). The mobile phase consisted of aqueous buffer solution as solvent A and acetonitrile/water (95:5, *v*/*v*) as solvent B. Gradient elution was performed from 5% B at 0 min to 95% B at 10 min, followed by a 5 min hold and 4 min equilibration. The flow rate was 0.4 mL/min, the injection volume was 2 μL, UV detection was performed at 254 nm, and the column temperature was maintained at 30 °C.

The pH of the aqueous mobile-phase component was adjusted to 2.5 for the acidic condition, 6.8 for the neutral condition, and 10.0 for the basic condition using 0.1% formic acid, 10 mM ammonium acetate, and 0.1% ammonium hydroxide, respectively. Retention times (tR) were recorded for all compounds under each condition. These experimentally measured values served as the target variable for subsequent modelling.

### 3.2. Datasets

Three datasets were used in this study, corresponding to chromatographic retention times measured under acidic, neutral, and basic reversed-phase LC conditions. Dataset1 contained 105 compounds measured under the acidic condition, dataset2 contained 117 compounds measured under the neutral condition, and dataset3 contained 112 compounds measured under the basic condition. Experimentally measured retention time was used as the target variable in each dataset.

For each compound, molecular structures were available and used as the basis for descriptor calculation. Although the datasets correspond to different chromatographic conditions, they were processed using the same descriptor generation workflow, enabling direct comparison of modelling strategies across conditions. For the pretrained GIN transfer learning benchmark, corresponding files containing compound identifiers, SMILES strings, and experimentally measured retention times were used to generate molecular graph representations for the same compounds.

Some compounds were present in more than one dataset, reflecting measurements of the same molecule under different chromatographic conditions. However, each dataset was treated independently for model development and evaluation, because each chromatographic system represents a separate condition-specific retention prediction problem.

The datasets were additionally characterized in terms of retention time distributions, molecular diversity, pairwise structural overlap, and broad structural and functional group composition. Dataset characterization was performed using RDKit 2026.03.3. Molecular structures were parsed from SMILES, and the largest organic fragment was retained as the parent structure when multiple disconnected components were present; formal charge, protonation state, and stereochemical information were otherwise retained. Canonical isomeric SMILES representations of the parent structures were used to determine exact structural overlap between datasets. Molecular diversity within each dataset was assessed using pairwise Tanimoto similarities calculated from radius-2, 2048-bit Morgan fingerprints. Broad structural and functional group categories were assigned using predefined SMARTS patterns. These categories were not mutually exclusive, and individual compounds could therefore be assigned to more than one category. Retention time distributions were summarized using the minimum, maximum, mean, standard deviation, median, and interquartile range. The complete aggregate characterization is provided in Tables S6–S8.

### 3.3. Molecular Descriptor Calculation and Preprocessing

Molecular descriptors were calculated using the Mordred descriptor package [14], which provides a broad set of two-dimensional and three-dimensional molecular descriptors, including constitutional, topological, geometrical, electronic, atom-type, autocorrelation, and physicochemical descriptor families. Descriptor calculation generated a high-dimensional feature matrix containing several hundred to thousands of candidate variables per compound.

To ensure numerical stability and reproducibility, a series of preprocessing steps was applied. First, non-numerical descriptors were removed. Descriptors containing missing values were excluded entirely rather than imputed in order to avoid introducing artificial correlations. Subsequently, descriptors with zero variance were removed.

Highly correlated descriptors were then filtered using an absolute pairwise Pearson correlation threshold of 0.95. This threshold was selected to remove only very strongly correlated and largely redundant descriptors while retaining descriptors that might contain complementary predictive information. Rácz et al. [30] systematically investigated molecular descriptor intercorrelation thresholds and found that relatively low thresholds could remove useful descriptors and impair model performance, whereas thresholds between 0.95 and 0.9999 were suitable for descriptor reduction, although the optimal value remained dataset-dependent. The value of 0.95 was therefore selected as the lower boundary of this recommended range, providing meaningful redundancy reduction without the more extensive descriptor removal associated with thresholds of 0.90 or 0.85. For each pair of descriptors with an absolute Pearson correlation coefficient greater than 0.95, one descriptor was removed. Importantly, zero-variance filtering and correlation filtering were fitted using the training data only during model validation. The resulting descriptor subset was then applied unchanged to the corresponding validation or test data. This procedure prevented information leakage from validation or test data into descriptor preprocessing. The original numeric Mordred descriptor matrices contained 1390, 1389, and 1387 descriptors for dataset1, dataset2, and dataset3, respectively. After training-fold-specific zero-variance and high-correlation filtering, the baseline models used 623.2 ± 4.4 descriptors for dataset1, 620.4 ± 4.7 descriptors for dataset2, and 612.2 ± 4.0 descriptors for dataset3.

### 3.4. Modelling Algorithms

A range of regression algorithms was evaluated to model the relationship between molecular descriptors and chromatographic retention time. Both linear and nonlinear approaches were included in order to assess whether retention was better described by relatively simple descriptor–response relationships or by more flexible nonlinear models.

Elastic Net regression [31] was included as a regularized linear model that combines L1 and L2 penalties. This approach is suitable for high-dimensional descriptor data because it can shrink coefficients, reduce the influence of weak variables, and handle correlated descriptors more effectively than ordinary least squares regression. Partial least squares (PLS) regression [32] was also evaluated as a chemometric reference method because it projects correlated descriptor variables into a smaller number of latent components. Nonlinear methods were included to capture more complex descriptor–retention relationships. Support vector regression (SVR) [33] was evaluated using both radial basis function and polynomial kernels. Tree-based ensemble methods, including Random Forest [34] and Extra Trees regression [35], were also tested because they can model nonlinear interactions and are relatively robust for heterogeneous descriptor spaces. k-nearest neighbours regression was included as an additional non-parametric reference method.

This set of algorithms provided a broad baseline against which the effect of explicit feature selection could be assessed.

### 3.5. Feature Selection Methods

Several feature selection approaches were evaluated to identify reduced descriptor subsets for chromatographic retention time prediction. Three categories of feature selection methods were considered: filter, embedded, and wrapper approaches.

Filter methods select descriptors using statistical criteria independently of the final predictive model. In this study, univariate feature selection was implemented using SelectKBest, where descriptors were ranked according to their individual association with retention time [17].

Embedded methods perform feature selection as part of model fitting. Elastic Net regression was used as an embedded feature selection approach because its L1/L2 regularization can shrink weak descriptor coefficients and retain a reduced set of variables with non-zero coefficients [31].

Wrapper methods evaluate descriptor subsets through repeated model fitting. Recursive feature elimination (RFE) was used as the wrapper-based strategy, where descriptors are iteratively removed according to their estimated importance until a selected subset size is reached [18]. RFE was implemented with different estimators, including Elastic Net, linear SVR, and Extra Trees, to assess whether the selected descriptor subsets depended on the underlying selection model.

All feature selection procedures were incorporated into the model training workflow and applied only within the training data of each validation fold. Feature selection, descriptor preprocessing, and hyperparameter optimization were therefore nested within cross-validation, preventing information from the validation or test data from influencing descriptor selection [36].

### 3.6. Nested Cross-Validation Strategy

QSRR model performance was evaluated using a nested cross-validation framework designed to provide an unbiased estimate of predictive performance during baseline modelling and feature selection assessment. The outer loop consisted of 10-fold cross-validation repeated with 10 different random seeds, resulting in 100 outer train–test evaluations for each dataset and modelling workflow. Within each outer training set, an inner cross-validation loop was used for hyperparameter optimization and, where applicable, feature selection. This ensured that all model tuning, descriptor preprocessing and descriptor selection decisions were made using training data only, while the outer test folds remained independent.

Predictive performance was assessed using root mean squared error (RMSE), mean absolute error (MAE), and the coefficient of determination (R^2^), calculated on the outer test folds and aggregated across the 100 outer evaluations. R^2^ was calculated as:

(1) $$R^{2}=1-\frac{\sum_{i=1}^{n}{\left(y_{i}-{\hat{y}}_{i}\right)}^{2}}{\sum_{i=1}^{n}{\left(y_{i}-\bar{y}\right)}^{2}}$$

where $y_{i}$ is the experimental retention time, ${\hat{y}}_{i}$ is the predicted retention time, and $\bar{y}$ is the mean experimental retention time of the evaluation set. Under strict out-of-sample evaluation, such as the outer loop of nested cross-validation used in this work, $R^{2}$ may take negative values when the prediction error exceeds the variance of the test data. In that case, the model performs worse than a naïve predictor that assigns the mean test set retention time to all samples.

In addition to predictive performance, the frequency with which individual descriptors were selected across folds was recorded to assess feature stability.

### 3.7. Repeated External Validation

Following nested cross-validation, one representative optimized feature-selected QSRR workflow was retained for each dataset and evaluated using repeated pseudo-external validation. For each dataset, 100 random 80/20 train–test splits were generated using a fixed random seed. This validation design was used to assess model robustness across repeated local train–test partitions and to provide a direct matched comparison with pretrained GIN transfer learning.

For the feature-selected QSRR models, all descriptor preprocessing steps, including removal of zero-variance and highly correlated descriptors, were performed using the training subset only. Feature selection, hyperparameter optimization, and final model fitting were also performed using only the training data. The optimized model was then applied to the corresponding held-out test subset.

The final QSRR workflows selected for repeated external validation were RFE-Extra Trees/Extra Trees for dataset1, RFE-linear SVR/SVR-RBF for dataset2, and embedded Elastic Net for dataset3. The pretrained GIN transfer learning model was evaluated using the identical 100 repeated 80/20 train–test splits. For each split, the pretrained model was fine-tuned on the same local training compounds and evaluated on the same held-out test compounds. This enabled direct comparison between feature-selected QSRR and transfer learning under identical validation conditions.

Model performance was assessed using RMSE, MAE, R^2^, mean relative error, and median relative error. Relative error was calculated as:

(2) $${RE}_{i}\left(\%\right)=\left|\frac{y_{i}-{\hat{y}}_{i}}{y_{i}}\right|\times 100$$

where $y_{i}$ and ${\hat{y}}_{i}$ are the experimental and predicted retention times for compound i, respectively. Unless otherwise stated, repeated validation results are reported as mean ± standard deviation across the 100 train–test splits.

### 3.8. Y-Randomization

As a control for chance correlation, Y-randomization was performed for the selected dataset1 FS-QSRR workflow, RFE-Extra Trees/Extra Trees. The analysis included one run using the original retention time response and 100 runs in which the response vector was independently permuted. The same 105 compounds and Mordred descriptor matrix were used in every run.

For the true response run and every randomized response run, the complete leakage-safe modelling workflow was repeated. This included training-fold-specific descriptor preprocessing, removal of zero-variance descriptors, removal of highly correlated descriptors using an absolute Pearson correlation threshold of 0.95, recursive feature elimination using Extra Trees, hyperparameter optimization, and fitting the final Extra Trees regression model. Each run used 10 outer cross-validation folds with an outer random seed of 0 and five-fold inner cross-validation.

Predictive performance was calculated from the pooled outer-fold predictions using RMSE, MAE, and $R^{2}$. For each metric, the empirical permutation probability was calculated as p=(1+b)/(1+N), where N=100 was the number of randomized response runs and b was the number of randomized models performing at least as well as the true response model. Lower RMSE and MAE and higher $R^{2}$ were considered better performance.

### 3.9. Pretrained GIN Transfer Learning Benchmark

To benchmark the feature-selected QSRR workflow against a contemporary graph-based deep learning approach, the pretrained GIN transfer learning model of Kwon et al. was evaluated [26]. The model was pretrained on the METLIN-SMRT retention time dataset and then fine-tuned separately on each local chromatographic dataset. The corresponding code and pretrained model weights were obtained from the authors’ public GitHub repository [37].

For each dataset, molecular structures were provided as SMILES strings [38,39] and converted into molecular graph representations using the preprocessing workflow supplied with the original repository. Molecules were parsed and sanitized using RDKit [40]. Atom-level features included atom type, formal charge, atom degree, hybridization state, number of attached hydrogens, total valence, hydrogen bond donor/acceptor status, chirality, ring size membership, aromaticity, and ring membership. Bond-level features included bond type, stereochemical state, ring membership, and conjugation.

All compounds were successfully converted into graph representations, giving 105, 117, and 112 valid molecular graphs for dataset1, dataset2, and dataset3, respectively. Molecular graph construction, atom and bond feature definitions, pretrained model weights, and fine-tuning procedures followed the original implementation of Kwon et al. The resulting graph datasets were saved in the compressed NumPy format required by the original implementation.

Transfer learning was performed independently for each chromatographic dataset. For each repeated validation split, a new GIN model was initialized, and the pretrained METLIN-SMRT model weights were loaded. The downstream prediction head was then reinitialized, while the pretrained weights of the GIN graph encoding layers were retained as the starting values. No network layers were frozen. Consequently, all trainable parameters in both the GIN graph encoder and the downstream prediction network were updated jointly during fine-tuning using only the compounds assigned to the local training set. Fine-tuning was performed using full-batch limited memory Broyden–Fletcher–Goldfarb–Shanno optimization with Huber loss [41] for up to 500 iterations. An $L_{2}$ parameter penalty of $1\times 10^{-5}$ was applied. Retention times were standardized using the mean and standard deviation of the local training set during model fitting, and predictions for the held-out compounds were transformed back to the original retention time scale before performance evaluation.

### 3.10. Matched Prediction Error Analysis

Because the feature-selected QSRR and pretrained GIN models were evaluated using identical repeated 80/20 train–test splits, predictions were matched directly by dataset, split number, compound identifier, and experimental retention time. For each matched prediction, absolute error was calculated as:

(3) $${AE}_{i}=\left|y_{i}-{\hat{y}}_{i}\right|$$

where $y_{i}$ and ${\hat{y}}_{i}$ are the experimental and predicted retention times for compound i, respectively. Error distributions were summarized using median absolute error, the 75th, 90th, and 95th percentile absolute errors, and the proportions of predictions exceeding absolute error thresholds of 1.0, 1.5, and 2.0 min. These statistics were used to determine whether differences between feature-selected QSRR and pretrained GIN transfer learning reflected broad improvements across the error distribution or were driven primarily by changes in the upper error tail. Split-level paired differences in RMSE, MAE, and $R^{2}$ were also calculated across the 100 repeated splits. This allowed the consistency of the GIN improvement to be assessed across repeated train–test partitions, rather than only from pooled prediction error summaries.

Statistical significance was assessed using the paired split-level RMSE, MAE, and $R^{2}$ values obtained from the identical 100 repeated random 80/20 train–test splits. For each dataset and performance metric, the GIN value from each split was paired with the corresponding FS-QSRR value from the same split. Paired differences were defined as the GIN value minus the corresponding FS-QSRR value; therefore, negative differences in RMSE and MAE and positive differences in $R^{2}$ favoured GIN. The paired differences were evaluated using two-sided Wilcoxon signed-rank tests. Because nine comparisons were performed across three datasets and three performance metrics, the resulting *p*-values were adjusted using the Holm procedure. Holm-adjusted *p*-values below 0.05 were considered statistically significant. Because repeated random splits reused compounds across different training and test partitions, the resulting split-level observations were not fully independent. The statistical tests were therefore interpreted as measures of the consistency of the paired differences within the repeated-validation procedure. Further details and complete results are provided in Supplementary Table S4.

### 3.11. Hyperparameter Optimization

Hyperparameter optimization was performed within the inner loop of the nested cross-validation procedure for all QSRR models. This ensured that model selection, feature selection tuning and descriptor preprocessing were carried out exclusively using the training data available within each outer fold, preventing information leakage into the held-out test data.

For the baseline regression models evaluated without explicit feature selection, hyperparameters were optimized using randomized search. Model-specific parameter distributions and ranges are listed in Supplementary Information Table S1. For each outer training split, candidate hyperparameter combinations were sampled from these distributions and evaluated within the inner cross-validation loop. The combination giving the lowest inner-loop RMSE was then selected and used to fit the final model for that outer split. Randomized search was used to efficiently explore broad hyperparameter spaces, particularly for models with continuous parameters such as SVR and Elastic Net.

For the feature selection workflows, hyperparameters were optimized using exhaustive grid search over the predefined grids shown in Supplementary Information Table S2. In these workflows, the search space included both feature selection parameters, such as the number of retained descriptors, and final model parameters, such as regularization strength, tree model parameters, or SVR kernel parameters. For the RFE-Linear SVR/SVR-RBF workflow, linear SVR was used as the recursive feature elimination estimator, whereas SVR-RBF was used as the final predictive model. The grid search procedure therefore optimized the descriptor subset and predictive model jointly within the inner cross-validation loop.

In all cases, the held-out outer test data were used only for final performance evaluation. This fully nested design ensured that reported performance values reflected out-of-sample predictive performance rather than information leakage from model selection or descriptor selection.

### 3.12. Computational Details

All modelling and analysis workflows were implemented in Python and executed on a high-performance computing cluster using SLURM job arrays. Separate computational environments were used for the descriptor-based workflow and the pretrained GIN transfer learning benchmark because the two workflows required different software dependencies.

The feature-selected QSRR workflow was performed using Python 3.10 with scikit-learn 1.7.2, NumPy 1.26.4, pandas 2.1.4, and Mordred 1.2.0. Baseline modelling, nested cross-validation, feature selection benchmarking, repeated external validation, descriptor frequency analysis and matched error analysis were implemented using scikit-learn pipelines and custom Python scripts.

The pretrained GIN transfer learning benchmark was performed in a separate Python environment using Python 3.10, PyTorch 2.0.1 with CUDA 11.8 support, DGL 1.1.2, RDKit 2023.03.2, NumPy 1.23.5, pandas 1.5.2, SciPy 1.9.3, and scikit-learn 1.2.0. Transfer learning calculations were executed on the GPU partition of the cluster, with one GPU allocated per split-level task.

Intermediate outputs, including split-level predictions, split-level performance metrics, selected descriptor lists, graph datasets, matched prediction error tables, and error distribution summaries, were retained for downstream analysis and reproducibility.

**Figure 3:**
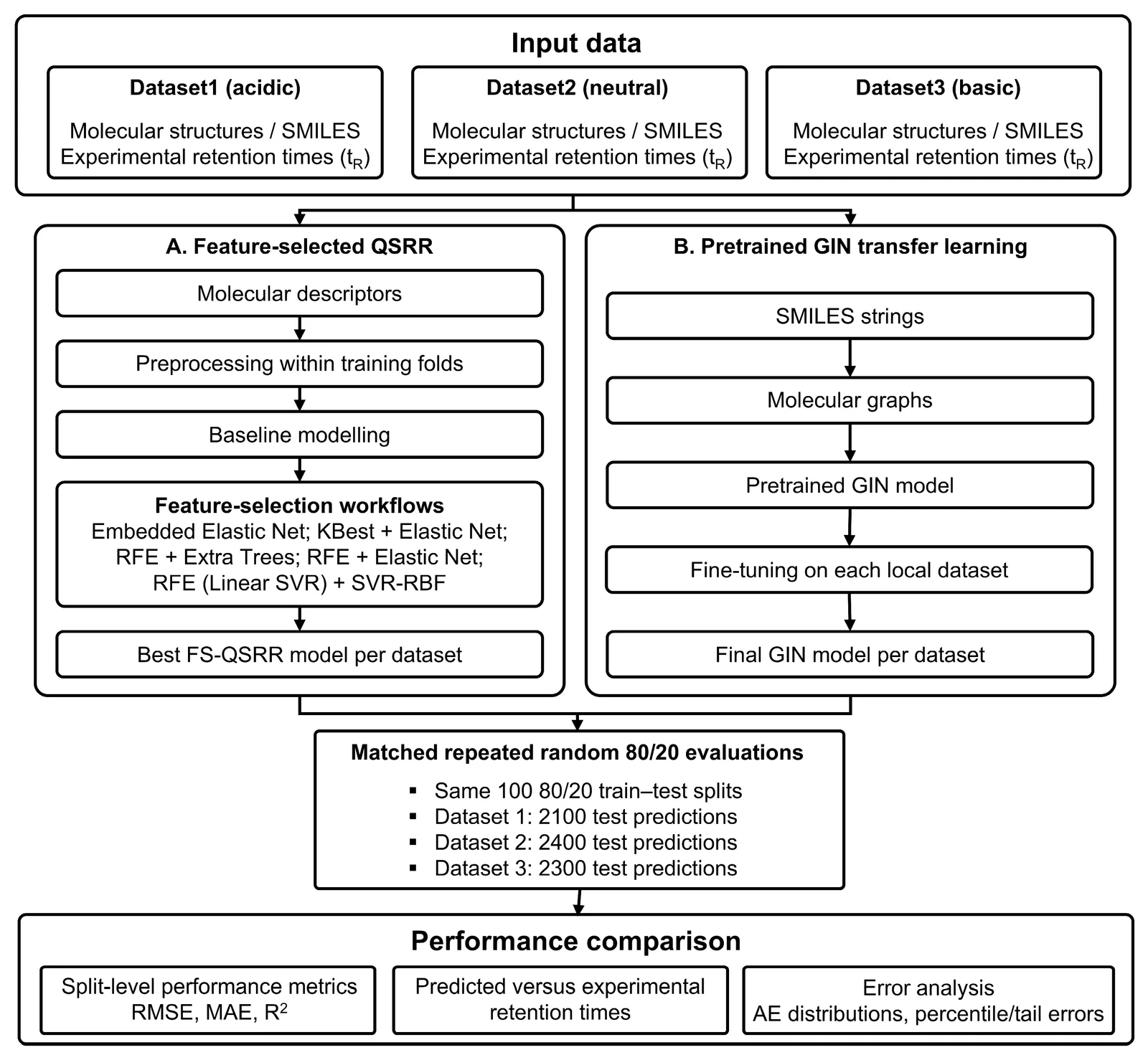
Overall workflow for the development and matched comparison of feature-selected quantitative structure–retention relationship (FS-QSRR) models and pretrained graph isomorphism network (GIN) transfer learning across three reversed-phase liquid chromatography datasets measured under acidic, neutral, and basic conditions. In the FS-QSRR branch, molecular descriptors were subjected to training-fold-specific preprocessing, baseline modelling, and feature selection workflows, followed by selection of one representative FS-QSRR model for each dataset. In the GIN branch, SMILES strings were converted into molecular graphs and used to fine-tune the pretrained GIN separately for each local dataset. The selected FS-QSRR and final GIN models were evaluated using the same 100 repeated random 80/20 train–test splits, producing 2100, 2400, and 2300 matched test predictions for dataset1, dataset2, and dataset3, respectively. Performance was compared using split-level RMSE, MAE, and R^2^, predicted versus experimental retention times, and absolute error distributions and upper-tail error statistics. Arrows indicate the sequence of data processing, model development, validation, and comparison.

The overall modelling and validation workflow is summarized in Figure 3. In brief, descriptor-based QSRR models were first evaluated without explicit feature selection, followed by feature selection benchmarking and selection of one representative FS-QSRR workflow for each dataset. These selected workflows were then compared with pretrained GIN transfer learning using identical repeated random 80/20 train–test splits, enabling matched comparison of split-level performance metrics and pooled prediction error distributions.

## 4. Conclusions

This study compared feature-selected QSRR and pretrained graph isomorphism network transfer learning for condition-specific retention time prediction using three datasets representing acidic, neutral, and basic chromatographic conditions. FS-QSRR combined Mordred descriptors with several feature selection and regression strategies, whereas the GIN approach used molecular graphs and transferred representations learned during large-scale pretraining. Under matched validation conditions, the pretrained GIN generally provided greater predictive accuracy and fewer large prediction errors, while FS-QSRR enabled descriptor-level interpretation and revealed differences in the molecular properties associated with retention under the three chromatographic conditions.

The choice between the two approaches should depend on the intended application. Pretrained graph-based transfer learning is particularly attractive when predictive accuracy is the principal objective and an appropriate pretrained model, sufficient local calibration data, and the necessary computational resources are available. FS-QSRR remains valuable when transparency, simpler implementation, model parsimony, and physicochemical interpretation are important. Feature selection should not be expected to improve predictive accuracy automatically, particularly for small datasets containing correlated and partly redundant descriptors. Its main benefits may instead lie in simplifying the descriptor representation and identifying molecular properties that are consistently associated with retention. FS-QSRR and pretrained GIN should therefore be considered complementary approaches rather than direct substitutes.

The conclusions are limited to the chemical and chromatographic spaces represented by the investigated datasets. Neither the selected descriptor sets nor the fitted models should be assumed to transfer unchanged to different analyte classes or chromatographic systems. Future work will extend the comparison to additional condition-specific datasets and investigate how the structural similarity between predicted compounds and the available training data influences the accuracy of FS-QSRR and GIN predictions. This should support a more informed assessment of model applicability and transferability across chemical space.

## Figures and Tables

**Table 1:** Baseline performance of regression models evaluated without explicit feature selection using nested cross-validation. Values represent mean ± standard deviation across 100 outer test evaluations generated from 10 outer folds repeated with 10 random seeds. Hyperparameters were optimized within the inner cross-validation loop for each outer training split.

Dataset	Model	RMSE	MAE	R^2^
		(Mean ± SD)	(Mean ± SD)	
	Extra Trees	0.759 ± 0.216	0.576 ± 0.163	0.656 ± 0.158
	Elastic Net	0.796 ± 0.236	0.631 ± 0.177	0.607 ± 0.264
	SVR-poly	0.870 ± 0.265	0.659 ± 0.193	0.517 ± 0.338
dataset1	Random Forest	0.896 ± 0.241	0.713 ± 0.184	0.519 ± 0.232
(acidic pH)	SVR-RBF	0.945 ± 0.285	0.719 ± 0.213	0.464 ± 0.269
	Linear-SVR	0.956 ± 0.416	0.697 ± 0.248	0.343 ± 0.779
	PLS	1.001 ± 0.371	0.744 ± 0.237	0.304 ± 0.661
	KNN	1.064 ± 0.250	0.824 ± 0.210	0.290 ± 0.389
	Extra Trees	0.781 ± 0.178	0.617 ± 0.145	0.616 ± 0.486
	SVR-RBF	0.794 ± 0.208	0.616 ± 0.156	0.611 ± 0.399
	Random Forest	0.809 ± 0.195	0.653 ± 0.152	0.576 ± 0.563
dataset2	SVR-poly	0.810 ± 0.228	0.626 ± 0.171	0.565 ± 0.596
(neutral pH)	Linear SVR	0.818 ± 0.275	0.644 ± 0.186	0.567 ± 0.470
	Elastic Net	0.834 ± 0.225	0.664 ± 0.145	0.573 ± 0.372
	PLS	0.910 ± 0.276	0.708 ± 0.180	0.450 ± 0.615
	KNN	0.938 ± 0.252	0.729 ± 0.185	0.457 ± 0.529
	Elastic Net	0.916 ± 0.259	0.711 ± 0.212	0.639 ± 0.223
	SVR-RBF	1.008 ± 0.214	0.787 ± 0.198	0.578 ± 0.195
	SVR-poly	1.027 ± 0.281	0.793 ± 0.236	0.550 ± 0.261
dataset3	Extra Trees	1.067 ± 0.233	0.868 ± 0.200	0.519 ± 0.263
(basic pH)	Linear-SVR	1.077 ± 0.257	0.841 ± 0.213	0.511 ± 0.253
	PLS	1.164 ± 0.289	0.920 ± 0.237	0.425 ± 0.330
	Random Forest	1.177 ± 0.229	0.982 ± 0.216	0.429 ± 0.250
	KNN	1.335 ± 0.268	1.087 ± 0.246	0.258 ± 0.324

**Table 2:** Performance of feature selection strategies evaluated using nested cross-validation. Feature selection and model hyperparameters were optimized jointly by exhaustive grid search within the inner cross-validation loop. Values represent mean ± standard deviation across 100 outer test evaluations generated from 10 outer folds repeated with 10 random seeds. The average number of selected features is reported across the same outer evaluations. In the RFE-Linear SVR/SVR-RBF workflow, linear SVR was used as the recursive feature elimination estimator, whereas SVR-RBF was used as the final predictive model.

Dataset	FS Method	Model	RMSE	MAE	R^2^	Avg. # Features
			(Mean ± SD)	(Mean ± SD)		
dataset1 (acidic pH)	Embedded Elastic Net	Elastic Net	0.796 ± 0.236	0.631 ± 0.177	0.607 ± 0.264	52.74
	SelectKBest	Elastic Net	0.811 ± 0.201	0.647 ± 0.157	0.594 ± 0.210	490.12
	RFE-Elastic Net	Elastic Net	0.820 ± 0.226	0.643 ± 0.178	0.570 ± 0.305	46.80
	RFE-Extra Trees	Extra Trees	0.770 ± 0.209	0.585 ± 0.151	0.639 ± 0.178	14.80
	RFE-Linear SVR	SVR-RBF	0.835 ± 0.216	0.639 ± 0.168	0.561 ± 0.256	27.15
dataset2 (neutral pH)	Embedded Elastic Net	Elastic Net	0.834 ± 0.225	0.664 ± 0.145	0.573 ± 0.372	72.75
	SelectKBest	Elastic Net	0.799 ± 0.197	0.641 ± 0.155	0.610 ± 0.341	194.35
	RFE-Elastic Net	Elastic Net	0.821 ± 0.285	0.652 ± 0.162	0.536 ± 0.753	69.60
	RFE-Extra Trees	Extra Trees	0.794 ± 0.192	0.629 ± 0.158	0.599 ± 0.477	39.30
	RFE-Linear SVR	SVR-RBF	0.792 ± 0.216	0.604 ± 0.154	0.606 ± 0.387	43.70
dataset3 (basic pH)	Embedded Elastic Net	Elastic Net	0.916 ± 0.259	0.711 ± 0.212	0.639 ± 0.223	68.90
	SelectKBest	Elastic Net	0.938 ± 0.263	0.730 ± 0.218	0.615 ± 0.259	540.11
	RFE-Elastic Net	Elastic Net	0.945 ± 0.271	0.736 ± 0.213	0.606 ± 0.259	53.90
	RFE-Extra Trees	Extra Trees	1.040 ± 0.257	0.824 ± 0.205	0.536 ± 0.276	22.75
	RFE-Linear SVR	SVR-RBF	0.951 ± 0.264	0.726 ± 0.226	0.612 ± 0.233	43.10

**Table 3:** Direct comparison of the best feature-selected QSRR workflow and pretrained GIN transfer learning using matched repeated 80/20 validation. Values represent mean ± standard deviation across 100 matched random train–test splits. For each dataset, FS-QSRR and GIN were evaluated on identical held-out test compounds in each split. The repeated 80/20 design generated 2100, 2400, and 2300 held-out test predictions for dataset1, dataset2, and dataset3, respectively.

Dataset	Model/Workflow	RMSE	MAE	R^2^	Mean RE (%)	Median RE (%)
dataset1 (acidic pH)	RFE-Extra Trees/Extra Trees	0.816 ± 0.141	0.604 ± 0.102	0.659 ± 0.105	13.047 ± 2.789	8.988 ± 2.069
	Pretrained GIN	0.613 ± 0.143	0.479 ± 0.119	0.802 ± 0.111	10.479 ± 3.276	7.773 ± 2.384
dataset2 (neutral pH)	RFE-linear SVR/SVR-RBF	0.868 ± 0.162	0.664 ± 0.126	0.618 ± 0.159	11.275 ± 2.563	8.656 ± 2.421
	Pretrained GIN	0.665 ± 0.111	0.518 ± 0.087	0.773 ± 0.102	8.688 ± 1.635	6.892 ± 1.690
dataset3 (basic pH)	Embedded Elastic Net	1.019 ± 0.187	0.766 ± 0.134	0.619 ± 0.169	11.041 ± 2.434	7.950 ± 2.268
	Pretrained GIN	0.876 ± 0.126	0.687 ± 0.107	0.721 ± 0.099	10.031 ± 2.003	7.356 ± 2.054

**Table 4:** Distribution of absolute prediction errors for best feature-selected QSRR workflows and pretrained GIN transfer learning. Values were calculated from pooled matched test set predictions across the 100 repeated 80/20 splits. AE = absolute error.

Dataset	Model/Workflow	Median AE	75th Percentile AE	90th Percentile AE	95th Percentile AE	AE >1.0 min	AE >1.5 min	AE >2.0 min
dataset1 (acidic pH)	RFE-Extra Trees/Extra Trees	0.419	0.848	1.427	1.739	19.7%	9.0%	3.0%
	Pretrained GIN	0.378	0.688	1.032	1.246	11.2%	2.0%	0.6%
dataset2 (neutral pH)	RFE-Linear SVR/SVR-RBF	0.516	0.898	1.471	1.893	20.5%	9.7%	3.8%
	Pretrained GIN	0.419	0.741	1.060	1.272	12.4%	2.9%	1.0%
dataset3 (basic pH)	Embedded Elastic Net	0.568	1.058	1.739	2.176	27.0%	14.5%	6.5%
	Pretrained GIN	0.546	1.054	1.473	1.766	27.3%	9.4%	2.4%

## Data Availability

The data and modelling code supporting the findings of this study are available from the corresponding author upon reasonable request. The data are not publicly available because some of the molecular structures are proprietary and are subject to third-party confidentiality restrictions. Access to the restricted data may therefore require permission from the relevant data owner.

## Supplementary Materials

The following supporting information can be downloaded at https://www.mdpi.com/article/10.3390/ijms27167164/s1.
