# Supplementary material for: Condition-Specific LC Retention Time Prediction: Feature-Selected QSRR Versus Pretrained Graph Isomorphism Network Transfer Learning
Source: Int J Mol Sci. 2026 Aug 11;27(16):7164. doi: 10.3390/ijms27167164 (PMC13513519; doi:10.3390/ijms27167164)
Supplement: Supplementary file 1 [file ijms-27-07164-s001.zip › ijms-4455466-supplementary.pdf]

Supplementary Materials

Condition-Specific LC Retention-Time Prediction: Feature-Selected QSRR versus Pretrained Graph Isomorphism Network Transfer Learning

Roman Szucs [1,*], Emília Sýkorová [1], Iveta Boháčová [1], Ilaria Neri [2], Lucy M. Morgan [3], Merisa Moriarty [4], Melissa Hanna-Brown [2]

1 Department of Analytical Chemistry, Faculty of Natural Sciences, Comenius University Bratislava, Ilkovičova 6, SK-84215 Bratislava, Slovakia; sykorova132@uniba.sk (E.S.); iveta.hukelova4@uniba.sk (I.B.)
2 School of Chemistry, University College Cork, College Road, T12 YN60 Cork, Ireland; ineri@ucc.ie (I.N.); mhanna-brown@ucc.ie (M.H.-B.)
3 Pfizer Worldwide Research, Development and Medical, Sandwich CT13 9NJ, UK; lucy.morgan@pfizer.com
4 Pfizer Worldwide Research, Development and Medical, Ringaskiddy, P43 X336 Cork, Ireland; merisa.moriarty@pfizer.com
* Correspondence: roman.szucs@uniba.sk

Contents

Table S1. Hyperparameter distributions used for randomized search in baseline regression models evaluated without explicit feature selection. Hyperparameters were optimized within the inner cross-validation loop of the nested validation framework. For each outer split, randomized search sampled candidate hyperpatameter combinations from the distributions or discrete sets shown below, and the model minimizing inner-loop RMSE was selected.

Table S2. Hyperparameter grids used for feature-selection workflows. Feature-selection parameters and model hyperparameters were optimized jointly by exhaustive grid search within the inner cross-validation loop. In the RFE-linear SVR / SVR-RBF workflow, linear SVR was used only as the recursive feature-elimination estimator, whereas SVR-RBF was used as the final predictive model.

Table S3. Consolidated descriptor-frequency table for robust and manuscript-discussed descriptors identified across feature-selection workflows.

Table S4. Paired statistical comparison of FS-QSRR and pretrained GIN transfer learning across 100 matched repeated random 80/20 train–test splits.

Table S5. Y-randomization results for the selected Dataset1 FS-QSRR workflow, RFE-Extra Trees / Extra Trees. The true-response result was compared with the results obtained from 100 independent randomizations of the retention-time response.

Table S6. Retention-time distributions and molecular-similarity characteristics of the three condition-specific datasets.

Table S7. Pairwise overlap of parent structures between the three condition-specific datasets.

Table S8. Broad structural and functional-group composition of the three condition-specific datasets.

Table S1. Hyperparameter distributions used for randomized search in baseline regression models evaluated without explicit feature selection. Hyperparameters were optimized within the inner cross-validation loop of the nested validation framework. For each outer split, randomized search sampled candidate hyperpatameter combinations from the distributions or discrete sets shown below, and the model minimizing inner-loop RMSE was selected.

**Model**	**Hyperparameter**	**Distribution / Range**
Elastic Net	α	log-uniform ($10^{-4} – 10^{2}$)
	l_1 ratio	uniform (0 – 1)
linear SVR	C	log-uniform ($10^{-2} – 10^{3}$)
	ε	log-uniform (10^{-3} – 1)
SVR-RBF	C	log-uniform ($10^{-2} – 10^{3}$)
	γ	log-uniform ($10^{-4} – 10^{1}$)
	ε	log-uniform (10^{-3} – 1)
SVR-poly	C	log-uniform ($10^{-2} – 10^{3}$)
	degree	{2, 3, 4}
	γ	{scale, auto}
	coef0	uniform (0 – 3)
	ε	log-uniform (10^{-3} – 1)
Random Forest	n_estimators	integer (200 – 1000)
	max_depth	{None, 3, 5, 7, 10, 15, 20}
	min_samples_split	integer (2 – 10)
	min_samples_leaf	integer (1 – 5)
	max_features	{1.0, sqrt, log2}
Extra Trees	n_estimators	integer (200 – 1000)
	max_depth	{None, 3, 5, 7, 10, 15, 20}
	min_samples_split	integer (2 – 10)
	min_samples_leaf	integer (1 – 5)
	max_features	{1.0, sqrt, log2}
PLS	n_components	integer (1 – 15)
KNN	n_neighbors	integer (1 – 15)
	weights	{uniform, distance}
	p	{1, 2}

Table S2. Hyperparameter grids used for feature-selection workflows. Feature-selection parameters and model hyperparameters were optimized jointly by exhaustive grid search within the inner cross-validation loop. In the RFE-linear SVR / SVR-RBF workflow, linear SVR was used only as the recursive feature-elimination estimator, whereas SVR-RBF was used as the final predictive model.

**Feature-selection workflow**	**Component**	**Hyperparameter**	**Values**
Embedded Elastic Net	Elastic Net model	α	0.001, 0.01, 0.1, 1, 10
		l_1 ratio	0.1, 0.3, 0.5, 0.7, 0.9
SelectKBest + Elastic Net	SelectKBest selector	k	5, 10, 15, 20, 30, 40, 50, 75, 100, all
	Elastic Net model	α	0.001, 0.01, 0.1, 1, 10
		l_1 ratio	0.1, 0.3, 0.5, 0.7, 0.9
RFE-Elastic Net / Elastic Net	RFE selector	n_features_to_select	5, 10, 20, 30, 40, 50, 75, 100
	RFE Elastic Net estimator	α	0.001, 0.01, 0.1, 1
		l_1 ratio	0.1, 0.5, 0.9
	Final Elastic Net model	α	0.001, 0.01, 0.1, 1, 10
		l_1 ratio	0.1, 0.3, 0.5, 0.7, 0.9
RFE-Extra Trees / Extra Trees	RFE selector	n_features_to_select	5, 10, 20, 30, 40, 50, 75, 100
	Final Extra Trees model	n_estimators	200, 400
		max_features	sqrt, 0.3, 0.5
		min_samples_leaf	1, 2, 4
RFE-linear SVR / SVR-RBF[1)]	RFE selector	n_features_to_select	5, 10, 20, 30, 40, 50
	Linear SVR RFE estimator	C	0.1, 1, 10
	Final SVR-RBF model	C	0.1, 1, 10, 100
		γ	scale, 0.01, 0.1, 1
		ε	0.01, 0.1, 0.2

Table S3. Consolidated descriptor-frequency table for robust and manuscript-discussed descriptors identified across feature-selection workflows.

Dataset	Condition	Descriptor	Mean selection frequency	Workflows with frequency ⩾ 0.50	Embedded Elastic Net	SelectKBest / Elastic Net	RFE-Elastic Net	RFE-Extra Trees	RFE-linear SVR / SVR-RBF
dataset1	acidic pH	nBase	1.000	5	1.000	1.000	1.000	1.000	1.000
dataset1	acidic pH	AMID_N	0.984	5	1.000	1.000	1.000	1.000	0.920
dataset1	acidic pH	SLogP	0.916	5	1.000	1.000	0.970	1.000	0.610
dataset1	acidic pH	PEOE_VSA13	0.915	4	1.000	0.780	0.900	0.000	0.980
dataset1	acidic pH	MATS6i	0.820	4	0.920	0.800	0.820	0.000	0.740
dataset1	acidic pH	NaaN	0.798	4	0.980	0.960	0.700	0.970	0.380
dataset1	acidic pH	SdssC	0.755	4	0.940	0.780	0.680	0.000	0.620
dataset1	acidic pH	n6aHRing	0.706	4	0.780	0.830	0.750	0.680	0.490
dataset1	acidic pH	n8FRing	0.705	3	0.910	0.770	0.730	0.000	0.410
dataset1	acidic pH	AMID_X	0.690	3	0.960	0.880	0.550	0.000	0.370
dataset1	acidic pH	nBr	0.675	3	0.920	0.780	0.720	0.000	0.280
dataset1	acidic pH	ETA_dEpsilon_D	0.652	4	0.940	0.780	0.740	0.130	0.670
dataset1	acidic pH	SIC2	0.652	3	0.940	0.780	0.720	0.000	0.170
dataset1	acidic pH	RPCG	0.644	4	0.630	0.780	0.840	0.030	0.940
dataset1	acidic pH	BCUTdv-1l	0.637	4	0.510	0.780	0.710	0.000	0.550
dataset1	acidic pH	BCUTZ-1l	0.636	3	0.990	0.840	0.760	0.250	0.340
dataset1	acidic pH	EState_VSA5	0.606	4	0.760	0.780	0.650	0.060	0.780
dataset1	acidic pH	PEOE_VSA3	0.527	3	0.230	0.780	0.600	0.000	0.500
dataset2	neutral pH	SLogP	0.880	4	1.000	1.000	0.950	1.000	0.450
dataset2	neutral pH	ETA_dEpsilon_D	0.860	5	1.000	1.000	0.770	0.890	0.640
dataset2	neutral pH	AMID_N	0.792	4	0.990	0.990	0.710	0.940	0.330

dataset2	neutral pH	ATS2d	0.713	3	0.320	1.000	0.600	0.930	0.000
dataset2	neutral pH	ATSC0i	0.705	3	0.330	1.000	0.620	0.870	0.000
dataset2	neutral pH	FilterItLogS	0.692	4	0.640	1.000	0.710	0.910	0.200
dataset2	neutral pH	n6Ring	0.593	3	0.740	1.000	0.570	0.060	0.000
dataset2	neutral pH	BCUTZ-1l	0.590	3	0.930	0.940	0.100	0.060	0.920
dataset2	neutral pH	MDEC-23	0.586	3	0.320	1.000	0.630	0.970	0.010
dataset2	neutral pH	VE2_A	0.586	3	0.550	1.000	0.490	0.630	0.260
dataset2	neutral pH	GGI6	0.570	3	0.300	0.860	0.540	0.580	0.000
dataset2	neutral pH	ATS3Z	0.568	3	0.920	0.980	0.790	0.140	0.010
dataset2	neutral pH	nX	0.558	4	0.570	0.800	0.530	0.760	0.130
dataset2	neutral pH	AMID_h	0.530	4	0.500	0.780	0.130	0.600	0.640
dataset2	neutral pH	nC	0.508	3	0.360	0.750	0.650	0.750	0.030
dataset2	neutral pH	PEOE_VSA6	0.504	3	0.940	0.870	0.130	0.080	0.500
dataset3	basic pH	nAcid	1.000	5	1.000	1.000	1.000	1.000	1.000
dataset3	basic pH	nSpiro	0.927	4	0.950	0.860	0.940	0.000	0.960
dataset3	basic pH	ETA_dEpsilon_D	0.918	5	1.000	1.000	0.940	0.820	0.830
dataset3	basic pH	n10FaRing	0.870	4	0.980	0.860	0.790	0.000	0.850
dataset3	basic pH	AATSC1c	0.852	5	0.990	1.000	0.720	0.940	0.610
dataset3	basic pH	AATSC0c	0.840	4	1.000	1.000	0.980	0.240	0.980
dataset3	basic pH	Xc-4dv	0.838	4	0.740	0.860	0.810	0.000	0.940
dataset3	basic pH	FilterItLogS	0.836	5	0.980	1.000	0.670	1.000	0.530
dataset3	basic pH	EState_VSA4	0.802	4	0.620	0.920	0.800	0.000	0.870
dataset3	basic pH	BCUTc-1l	0.796	4	0.950	0.860	0.860	0.410	0.900
dataset3	basic pH	ATSC3dv	0.782	4	0.920	0.860	0.710	0.000	0.640
dataset3	basic pH	BCUTZ-1l	0.762	4	0.880	1.000	0.810	0.400	0.720
dataset3	basic pH	NssO	0.742	4	1.000	0.870	0.870	0.300	0.670
dataset3	basic pH	ATSC3s	0.735	3	0.980	0.860	0.610	0.000	0.490
dataset3	basic pH	PEOE_VSA2	0.733	4	0.790	0.860	0.720	0.000	0.560
dataset3	basic pH	SLogP	0.706	3	0.990	1.000	0.480	1.000	0.060

dataset3	basic pH	NddsN	0.700	3	0.860	0.860	0.670	0.000	0.410
dataset3	basic pH	nBondsT	0.682	4	0.830	0.760	0.610	0.000	0.530
dataset3	basic pH	ATSC1dv	0.680	3	0.810	1.000	0.400	0.000	0.510
dataset3	basic pH	EState_VSA5	0.668	4	1.000	0.960	0.580	0.020	0.780
dataset3	basic pH	BCUTd-1l	0.662	4	0.930	0.860	0.750	0.020	0.750
dataset3	basic pH	AATSC4c	0.624	3	0.970	1.000	0.360	0.630	0.160
dataset3	basic pH	BCUTi-1h	0.624	4	0.940	0.880	0.770	0.020	0.510
dataset3	basic pH	n10FaHRing	0.610	3	0.820	0.770	0.500	0.000	0.350
dataset3	basic pH	NssssN	0.603	3	0.360	0.780	0.580	0.000	0.690
dataset3	basic pH	BCUTare-1l	0.588	3	0.810	0.890	0.320	0.560	0.360
dataset3	basic pH	CIC1	0.558	3	0.750	1.000	0.100	0.910	0.030
dataset3	basic pH	BCUTc-1h	0.496	3	0.260	0.860	0.550	0.030	0.780
dataset3	basic pH	BCUTv-1l	0.486	3	0.520	0.860	0.450	0.060	0.540

Table S4. Paired statistical comparison of FS-QSRR and pretrained GIN transfer learning across 100 matched repeated random 80/20 train–test splits.

Dataset	Metric	FS-QSRR, mean ± SD	GIN, mean ± SD	Mean paired difference	Splits favoring GIN	Wilcoxon W	Raw p	Holm-adjusted p
Dataset1	RMSE	0.816 ± 0.141	0.613 ± 0.143	−0.203	93/100	205	1.50×10^{-15}	1.35×10^{-14}
Dataset1	MAE	0.604 ± 0.102	0.479 ± 0.119	−0.125	91/100	293	1.66×10^{-14}	1.16×10^{-13}
Dataset1	R^2	0.659 ± 0.105	0.802 ± 0.111	+0.143	93/100	228	2.84×10^{-15}	2.27×10^{-14}
Dataset2	RMSE	0.868 ± 0.162	0.665 ± 0.111	−0.204	89/100	310	2.62×10^{-14}	1.57×10^{-13}
Dataset2	MAE	0.664 ± 0.126	0.518 ± 0.087	−0.146	89/100	337	5.35×10^{-14}	2.14×10^{-13}
Dataset2	R^2	0.618 ± 0.159	0.773 ± 0.102	+0.155	89/100	327	4.11×10^{-14}	2.06×10^{-13}
Dataset3	RMSE	1.019 ± 0.187	0.876 ± 0.126	−0.143	85/100	505	3.77×10^{-12}	7.55×10^{-12}
Dataset3	MAE	0.766 ± 0.134	0.687 ± 0.107	−0.079	72/100	1017	2.16×10^{-7}	2.16×10^{-7}
Dataset3	R^2	0.619 ± 0.169	0.721 ± 0.099	+0.102	85/100	483	2.20×10^{-12}	6.61×10^{-12}

RMSE and MAE are expressed in minutes; $R^2$is dimensionless. Paired differences were calculated as GIN minus FS-QSRR. Negative paired differences in RMSE and MAE and positive paired differences in R^2therefore favour GIN. "Splits favouring GIN" indicates the number of matched splits in which GIN produced lower RMSE or MAE, or higher R^2, than FS-QSRR. $W$is the smaller of the positive and negative signed-rank sums from the two-sided Wilcoxon signed-rank test. Mean paired differences were calculated from the split-level values. Holm adjustment was performed across all nine dataset-by-metric comparisons.

Table S5. Y-randomization results for the selected Dataset1 FS-QSRR workflow, RFE-Extra Trees / Extra Trees. The true-response result was compared with the results obtained from 100 independent randomizations of the retention-time response.

Metric	True-response value	Randomized mean ± SD	Randomized median	Randomized range	Randomized models at least as good	Empirical permutation p
RMSE	0.781	1.493 ± 0.025	1.490	1.416–1.552	0/100	0.0099
MAE	0.582	1.252 ± 0.023	1.252	1.187–1.314	0/100	0.0099
R^2	0.711	−0.056 ± 0.035	−0.052	−0.142–0.050	0/100	0.0099

RMSE and MAE are reported in minutes; $R^2$is dimensionless. A randomized-response model was considered at least as good as the true-response model when its RMSE or MAE was less than or equal to the true-response value, or when its $R^2$was greater than or equal to the true-response value. Empirical permutation probabilities were calculated as $p = (1 + b)/(1 + N)$, where $b$is the number of randomized-response models performing at least as well as the true-response model and $N = 100$. The Y-randomization analysis used a dedicated 10-fold outer cross-validation evaluation and was separate from the 100 repeated 80/20 splits used for the final FS-QSRR–GIN comparison.

Table S6. Retention-time distributions and molecular-similarity characteristics of the three condition-specific datasets.

Dataset	Number of compounds	Retention-time range (min)	Mean retention time ± SD (min)	Median retention time (IQR) (min)	Mean pairwise Tanimoto ± SD	Median pairwise Tanimoto (IQR)
dataset1	105	2.31–8.58	5.157 ± 1.460	5.120 (4.020–6.320)	0.119 ± 0.076	0.105 (0.080–0.136)
dataset2	117	3.28–9.09	6.306 ± 1.459	6.540 (5.001–7.450)	0.118 ± 0.075	0.104 (0.080–0.134)
dataset3	112	3.64–11.05	7.564 ± 1.720	7.685 (5.998–8.975)	0.118 ± 0.078	0.104 (0.078–0.135)

Molecular similarity was calculated among unique parent structures using radius-2, 2048-bit Morgan fingerprints and the Tanimoto coefficient. Parent structures were defined as the largest organic fragment, while formal charge, protonation state, and stereochemical information were retained. SD, standard deviation; IQR, interquartile range.

Table S7. Pairwise overlap of parent structures between the three condition-specific datasets.

Dataset pair	Shared parent structures, n	Shared parent structures as percentage of the smaller dataset
dataset1–dataset2	88	83.8
dataset1–dataset3	83	79.0
dataset2–dataset3	95	84.8

Structural overlap was determined from canonical isomeric SMILES after selection of the largest organic fragment. The percentage of shared structures was calculated relative to the smaller dataset in each pairwise comparison.

Table S8. Broad structural and functional-group composition of the three condition-specific datasets.

Structural or functional category	dataset1, n (%)	dataset2, n (%)	dataset3, n (%)
Acyclic compound	3 (2.9)	0 (0.0)	2 (1.8)
Aromatic compound	92 (87.6)	109 (93.2)	99 (88.4)
Heteroaromatic compound	42 (40.0)	60 (51.3)	51 (45.5)
Non-aromatic ring system	57 (54.3)	66 (56.4)	67 (59.8)
Halogen-containing compound	38 (36.2)	43 (36.8)	45 (40.2)
Sulfur-containing compound	31 (29.5)	32 (27.4)	25 (22.3)
Phosphorus-containing compound	0 (0.0)	0 (0.0)	0 (0.0)
Carboxylic acid/carboxylate	18 (17.1)	19 (16.2)	17 (15.2)
Ester	17 (16.2)	12 (10.3)	17 (15.2)
Amide	43 (41.0)	51 (43.6)	49 (43.8)
Amine	53 (50.5)	63 (53.8)	57 (50.9)
Alcohol/phenol	31 (29.5)	36 (30.8)	35 (31.3)
Ether	44 (41.9)	44 (37.6)	46 (41.1)
Nitrile	1 (1.0)	1 (0.9)	1 (0.9)
Sulfonamide	20 (19.0)	21 (17.9)	15 (13.4)

Structural and functional-group categories were assigned using predefined SMARTS patterns. The categories were not mutually exclusive; therefore, an individual compound could be assigned to more than one category and the percentages within each dataset do not sum to 100%.
